# Hyperreactivity of Blood Leukocytes in Patients with NAFLD to *Ex Vivo* Lipopolysaccharide Treatment Is Modulated by Metformin and Phosphatidylcholine but Not by Alpha Ketoglutarate

**DOI:** 10.1371/journal.pone.0143851

**Published:** 2015-12-02

**Authors:** Agnieszka Zwolak, Agnieszka Szuster-Ciesielska, Jadwiga Daniluk, Olga Słabczyńska, Martyna Kandefer-Szerszeń

**Affiliations:** 1 Chair of Internal Medicine and Department of Internal Nursing, Medical University of Lublin, Lublin, Poland; 2 Department and Clinic of Endocrinology, Medical University of Lublin, Lublin, Poland; 3 Department and Clinic of Gastroenterology, Medical University of Lublin, Lublin, Poland; 4 Department of Virology and Immunology, Maria Curie-Sklodowska University, Lublin, Poland; University of Basque Country, SPAIN

## Abstract

**Introduction and Aims:**

Toll-like receptor 4 and proinflammatory cytokines play a central role in the progression of nonalcoholic fatty liver disease. We investigated IL-1, IL-6 and TNFα production and toll-like receptor 4 in both—obese and lean patients with non-alcoholic fatty liver disease who met different sets of metabolic syndrome criteria and linked the results with the disease burden.

**Materials and Methods:**

95 subjects were divided into four groups depending on the following criteria: presence or absence of metabolic syndrome and/or non-alcoholic fatty liver disease, glucose tolerance (prediabetes or normoglycemia) and BMI value (obese or lean). We determined the levels of IL-1β, IL-6, TNFα, and monocyte toll-like receptor 4 expression in fresh blood as well as in blood cultures treated with lipopolysaccharide with or without metformin, alphaketoglutarate or phosphatidylcholine supplementation.

**Results:**

The blood leukocytes of patients with non-alcoholic fatty liver disease are hypersensitive to lipopolysaccharide treatment and produce elevated levels of pro-inflammatory cytokines in response to *ex vivo* treatment with lipopolysaccharide. Moreover, they overexpress toll-like receptor-4. Hyperreactivity was typical mainly for obese patients with non-alcoholic fatty liver disease together with metabolic syndrome and decreased with the severity of disease. Metformin was the most effective in attenuation of hyperreactivity in all groups of patients with non-alcoholic fatty liver disease, but in obese patients the effectiveness of metformin was weaker than in lean. The reduction of cytokine level by metformin was accompanied by the decrease in toll-like receptor-4 expression. phosphatidylcholine also attenuated hyperreactivity to lipopolysaccharide but mainly in obese patients. Alpha ketoglutarate did not modulate cytokines’ level and toll-like receptor 4 expression in non-alcoholic fatty liver disease patients.

**Conclusions:**

Metformin and phosphatidylcholine attenuated lipopolysaccharide induced toll-like receptor 4 overexpression and overproduction of pro-inflammatory cytokines; however, their efficacy depended on combined presence of non-alcoholic fatty liver disease, metabolic syndrome and obesity.

## Introduction

Nonalcoholic fatty liver disease (NAFLD) is a condition in which excess fat accumulates in the liver of patient with no history of alcohol abuse or other causes for secondary hepatic steatosis. NAFLD is strongly associated with insulin resistance and metabolic syndrome (MS). NAFLD encompasses a histological spectrum of liver disease from simple steatosis through steatohepatitis (NASH) to fibrosis and ultimately cirrhosis. The majority of patients with NAFLD have simple steatosis that carries a relative benign prognosis [1]. Patients with NASH are at high risk of developing fibrosis, cirrhosis and hepatocellular carcinoma. Over 90% of patients with NAFLD have one or more features of MS and 30–40% of them fulfill criteria of MS including: increased waist circumference, impaired fasting glucose, hypertriglyceridemia, low serum high density lipoprotein cholesterol and hypertension. The severity of NAFLD is also associated with the severity of MS [1,2].

Obesity is strongly associated with NASH pathogenesis, largely related to changes in the serum concentration of several adipokines. In addition to obesity, chronic inflammation is another important contributing factor in NASH pathogenesis. Lipopolysaccharide (LPS) of gram-negative bacteria is considered a potent inducer of hepatic inflammation [3]. Moreover, low doses of LPS can causes hyperresponsivity to higher doses of LPS in experimental obese mice leading to accelerated NASH progression [3]. The pathogenesis of NASH remains unclear. It has been proposed that insulin resistance represents the first hit, leading to steatosis and that proinflammatory cytokines could cause a second hit, leading to NASH. Both adipokines such as leptin, adiponectin, visfatin and proinflammatory cytokines such as tumor necrosis factor α (TNFα), interleukin-6 (IL-6) and IL-1β could act as pathogenic factors in NASH development [4–7]. Recently, the role of Kupffer cells (KCs) as scavengers of LPS from blood in NAFLD is indicated. As the result of LPS recognition by Toll-like receptor 4 (TLR4) the perturbation in C-Jun-N-terminal kinase (JNK) and nuclear factor kappa B (NFkB) occurs in hepatocytes and leads to release of several proinflammatory cytokines and chemokines which promote steatosis of hepatocytes [8]. Such interplay between adipokines released by fat tissue and cytokines released mainly by hepatocytes leads finely to development of inflammation in the liver.

Metformin is the most widely used first-line therapy for type 2 diabetes (T2DM) and has numerous effects on human metabolism including improvements in endothelial dysfunction, homeostasis and oxidative stress, insulin resistance, lipid profiles and fat redistribution [9]. Metformin is also used for insulin resistance-related disease such as NAFLD. The results of recent *in vitro* studies show that metformin has a direct effect on inhibiting hepatocyte's and macrophage's inflammatory response stimulated by LPS leading to decrease in expression of IL1β, IL-6 and TNFα in macrophages [9].

Several studies suggest that low levels of hepatic phosphatidylcholine (PC) in the liver play role in the pathogenesis of NAFLD. PC supplementation in experimental mice deficient in phosphocholine cytidylyltransferase (which fed with high fat diet developed NASH within one week) protected them from steatosis but not prevented against the development of NASH [10]. In spite that PC is often used for treatment of liver steatosis in humans, its influence on liver inflammation is poorly examined. Another study revealed that PC treatment of obese mice fed with high fat diet alleviated hyperlipidemic changes and development of NAFLD [11]. A number of recent studies have demonstrated an anti-inflammatory potential of PC and its metabolites in various conditions linked to leukocyte activation, including ischemia, oxidative stress and endotoxin-induced injuries [12,13]. Moreover, in clinical practice, PC as part of normal diets, has been found to be effective as therapeutic agent.

Another substance which was proved to influence liver homeostasis disturbed by hepatotoxins is alpha ketoglutarate (AKG), free radical and ammonium ion scavenger, which can restore activities of ASPAT and ALAT in liver of mice treated with hepatotoxin N-nitrosodiethylamine back to the normal level [14,15]. Apart from the liver, in blood vessels of mice treated with AKG, glutathione peroxidase activity significantly increased and was followed by decreased total antioxidant status, therefore improving redox state and blood vessels elasticity in aged mice [16]. The results also indicate that AKG is effective in protecting from N-nitrosodiethylamine-induced perturbation in oxidative stress in many organs of rats [17]. AKG influences bone structure, amino acids and mineral absorption, muscle performance and even its show anticancer activity [18].

To examine the effect of metformin, PC and AKG on cytokines' production by leukocytes in subjects with NAFLD, we developed the *ex vivo* model of experiment in which blood samples diluted with medium were treated with mentioned above substances and induced to cytokines' production by treatment of 100 ng/ml of LPS. In order to detect the role of obesity and MS, we divided patients with NAFLD into 4 groups. First group included prediabetic obese patients with MS, second—lean patients without MS, third- obese patients without MS and forth—healthy individuals. The level of IL-1β, IL-6, TNF-α in sera of patients and in supernatants from cultures of blood cells treated with LPS were examined.

## Materials and Methods

### Study population

In total, 124 adults admitted to the Medical University Hospital in Lublin (Poland) participated in this study. Of 124 subjects we excluded those with the presence of different potential causes of liver disease: (a) seropositivity for HBsAg or anti-HCV antibody, (b) daily alcohol consumption over 20g, (c) treatment with hepatotoxic, steatosis-provoking, or immunosuppressive drugs during the previous 6 months, (d) Wilson’s disease or haemochromatosis. Other exclusion criteria were T2DM, malignancy, clinical atherosclerosis, hematological or chronic kidney diseases, active infection, metformin treatment and smoking. The control group comprised 22 healthy individuals undergoing a routine health check-up program. Informed written consent was obtained from all participants in this study, which was conducted according to the ethical principles stated in the Declaration of Helsinki and approved by the institutional review board at the Medical University of Lublin, Poland.

### Laboratory and abdominal ultrasonography (AU) assessment

From each individual, anthropometric data were recorded. Venous fasting blood samples were drawn from all the subjects to perform biochemical analyses, determine cytokine levels, measure TLR4 expressing monocytes, and develop ex vivo studies. The laboratory test included: glucose (mg/dl), HbA1C (%), total cholesterol (mg/dl) and its fractions (HDL, LDL, mg/dl), triglyceride (TG, mg/dl), aspartate aminotransferase (ASPAT, IU/L), alanine aminotransferase (ALAT, IU/L), high-sensitivity C-reactive protein (hs-CRP, mg/dl), and insulin (μl/ml). The degree of insulin resistance (IR) was determined by the homeostatic model assessment (HOMA-IR) with the formula: (glucose*insulin):22.5. Fatty liver was defined according to the results of AU: increased echogenicity of the hepatic parenchyma with an attenuation of the portal vein echogenicity. In six patients, the results of AU indicated advanced disease; therefore, liver biopsy was performed and such subjects were excluded from our study. We also calculated HSI and FLI indexes according to formulas: HSI = 8*ALAT/ASPAT ratio + BMI (+2 if female) [15], FLI = (e^0.953*loge (triglycerides) + 0.139*BMI + 0.718*loge (GGT) + 0.053*waist circumference – 15.745^)/(1 + e^0.953*loge (triglycerides) + 0.139*BMI + 0.718*loge (GGT) + 0.053*waist circumference – 15.745^) * 100 [19,20]. HSI and FLI<30.0 ruled out while HSI≥36.0 and FLI ≥ 60.0 ruled in hepatic steatosis detected by AU. However, the limitation of patient diagnosis was the lack of liver biopsy in all participants, which could reveal early fibrosis stage.

MS was defined using criteria of the International Diabetes Federation/National Heart, Lung and Blood Institute/American Heart Association (IDF/NHLBI/AHA-2009)—the presence of three or more of the following features: 1. waist circumference ≥94 cm in men or ≥80 cm in women; 2. triglyceride level ≥150 mg/dl; 3. HDL-cholesterol level <40 mg/dl in men and <50 mg/dl in women; 4. systolic blood pressure ≥130 mmHg or diastolic pressure ≥85 mmHg; 5. fasting plasma glucose level ≥100 mg/dl. Prediabetes was defined by a fasting glucose level of 100–125 mg/dl and/or HbA1C 5.7–6.4% (American Diabetes Association) [21]. The body mass index (BMI) (kg/m^2^) was calculated for each person. According to the BMI definition given by the World Health Organization, Caucasian patients with BMI 18.5–24.99 were included in the normal range, and those with BMI≥30.0 were classified as obese. Serum cytokine levels, IL-1β, IL-6, and TNFα were determined with commercially available kits (BD OptEIA, San Jose, CA, USA) according to manufacturer instructions. The minimum detectable concentration of IL-1β was 0.8 pg/ml, IL-6–2.2 pg/ml, and TNFα – 2 pg/ml.

Based on the IDF/NHLBI/AHA-2009 criteria of metabolic syndrome, results of AU supported by calculation of HSI and FLI indexes as well as BMI, we finally selected and divided 73 patients into 3 groups: group I–prediabetic, obese subjects with NAFLD and MS (meeting all IDF/NHLBI/AHA-2009 criteria) (n = 28); group II—lean patients with NAFLD and without MS (two of the five IDF/NHLBI/AHA-2009 criteria) (n = 23); group III–obese subjects without NAFLD and MS (one IDF/NHLBI/AHA-2009 criterion) (n = 22). Healthy individuals served as control group (IV) (n = 22).

### *Ex vivo* study design

We investigated production of IL-1β, IL-6, TNFα in sera, and TLR4 expression on monocytes of peripheral blood from the examined patients. Briefly, blood samples were collected into sterile heparynized tubes, diluted 1:5 with RPMI 1640 and cultured for 24 h with or without metformin (20 or 100 μM), AKG (10 or 25 mM) or PC (20 or 100 μg/ml) at 37°C and 5% CO_2_ with or without 100 ng/ml of LPS from *E*.*coli*. After 24 h each sample was centrifuged (300xg, 5 min) and culture supernatants were collected and frozen immediately at -80°C for no longer than three weeks until cytokines were measured (as specified above). Appropriate leukocyte pellets were stained with anti-TLR4 mAbs (see Flow cytometry). PC was manufactured by Sigma-Aldrich (Steinheim, Germany) (substance symbol: 8002-43-5). The quality and percentage composition of the lipids and the saturated to unsaturated fatty acids ratio in the PC product were compatible with the preparations used for treatment purposes (in accordance to United States Reference Standard). The PC concentrations used in the experiment (20–100 ug/ml) are equivalent with 25.8–129 microM range. The initial results concerning cytotoxicity of the examined substances were not included in the paper, but only nontoxic in vitro concentrations of PC were used in experiments. Moreover, the cytometry results seem to deny cytotoxicity. The remaining chemicals needed were purchased from Sigma-Aldrich (Steinheim, Germany), unless otherwise specified.

### Flow cytometry

100 μl of fresh heparynized blood or 100 μl of leukocyte pellets from *ex vivo* culture was stained with 10 μl of PE-anti-TLR4 mAbs (MCA2061PE) for 30 min in darkness (RT). Isotype-matched control antibody was used to detect nonspecific staining (MCA929PE) and mouse anti human CD14 mAbs–to monocytes gating strategy (MCA1568F). All antibodies were purchased form AbD Serotec, Düsseldorf, Germany. Then erythrocytes were lysed through incubation with the lysing solution (Becton Dickinson) for 10 min at RT, cells were washed twice with PBS containing 1% FCS and resuspended with 0.5 ml of 1% paraformaldehyde solution. Thirty thousand events were acquired (FACSCalibur, BD, Biosciences, Mountain View, CA) and monocytes were gated according to their characteristic FSC/SSC profiles compared with CD14 dot plot. TLR4 expression were analyzed with the CellQuestPro software, ver. 6.0, BD) and measured as MFI = MFI_TLR4_-MFI_isotype_.

### Data analysis

Normality of variables was tested with the Kolmogorov-Smirnov test. Normally distributed continuous variables were expressed as mean ± SD, and categorical variables were summarized as median with interquartile range. Quantitative variables with normal distribution were analyzed with one-way or two-way ANOVA with Tukey HSD as a post-hoc test. Comparisons between groups with categorical variables were evaluated by Kruskal-Wallis followed by Dunn test. Correlation analyses between continuous or categorical variables were performed by Pearson’s or Spearman’s, respectively. Data were analyzed by using STATISTICA software version 7.1 (StatSoft. Inc., Tulsa, OK, USA) and *P* value ≤ 0.05 was considered statistically significant.

## Results

### Anthropometric, metabolic, and biochemical characteristics of the subjects

Table 1 summarizes the general characteristics of the subjects included in our study. The mean age was similar in all groups, however, the groups I and II were dominated by males, 54% and 56% respectively, while III and IV–by women, 77% and 68% respectively. Other biochemical parameters and correlations between them were described in detail in our previous paper [22].

**Table 1:** Baseline clinical, anthropometric, and biochemical characteristics of the study populations.

Parameters	Group I	Group II	Group III	Group IV
	n = 28	n = 23	n = 22	n = 22
**Age** (years)	51.9±10.7	48.9±7.6	47.5±6.8	47.0±6.2
**Gender** (M:F)	15:13	13:10*	5:12*	7:15
**M:F** (%)	54:46	56:44	23:77	32:68
**Weight** (kg)	107.3±15.5*	70.5±11	108.9±10.8*	62.3±10.6
**BMI** (kg/m2)	36.5±5.8*	25.0±2.6	39.9±1.8	21.4±2.4
**Waist** (cm)				
**Male**	116.6±11.5*	91.2±2.2	110.7±13.6	88.6±1.1
**Female**	111.7±9.1*	76.9±6.2	112.3±12.0	72.5±5.6
**hs-CRP** (mg/dl)	7.33±2.8*	3.17±1.3	3.65±1.5	2.6±0.5
**ALAT** (IU/L)	80.67±18.75*	75.0±29.9*	21.6±3.9*	17.6±3.6
**ASPAT** (IU/L)	72.9±15.5*	68.0±12.0*	20.8±7.12*	16.5±3.7
**Glucose** (mg/dl)	103.9±4.8*	92.8±5.9	87.0±7.9	85.6±6.6
**Insulin** (μl/ml)	20.6±7.8*	15.4±2.6	19.9±2.7	8.2±1.8
**HbA1C** (%)	5.9±1.0*	4.58±0.47	4.62±0.38	4.4±0.32
**HOMA-IR**	5.2±1.5*	3.28±0.75	4.27±0.65	1.75±0.29
**C-peptide** (nmol/l)	4.62±1.6*	5.3±0.7*	3.9±0.9	3.08±0.6
**Total cholesterol** (mg/dl)	240.1±29.5*	213.3±26.9*	189.0±14.7*	178.3±6.5
**LDL-cholesterol** (mg/dl)	145.2±19.8*	111.3±18.2	99.2±11.4	93.3±6.8
**HDL-cholesterol** (mg/dl)	43.9±9.1*	46.1±6.9*	51.8±7.55	74.1±11.2
**Triglyceride** (mg/dl)	218.8±42.1*	185.0±35.5	145.3±2.3*	126.9±12.0
**Systolic BP** (mmHg)	136.9±7.2*	128.4±5.6	127.6±5.6	125.9±5.4
**Diastolic BP** (mmHg)	81.9±6.6*	77.1±5.4	75.9±4.8	76.9±5.1
**FLI**	95.7±4.7*	91.3±8.1*	60.8±9.9*	18.7±12.3
**HSI**	48.5±6.9*	45.5±5.2*	36.3±6.4*	28.4±1.8
**IL-1β** (pg/ml)	9.83±2.0*	4.45±1.92*	1.7±0.12*	1.23±0.52
**IL-6** (pg/ml)	6.72±1.75*	3.6±1.14	2.58±0.1	-
**TNFα** (pg/ml)	27.7±3.6*	15.5±1.4*	6.1±0.6*	3.02±0.5
**TLR4** (MFI)	53.19±6.7*	38.4±3.6*	27.1±5.6*	17.3±3.5

Group I–prediabetic, obese NAFLD with MS, group II—lean NAFLD without MS, group III—obese without MS, group IV—healthy individuals.

Data are expresses as means ± SD (normally distributed continuous variables).

*statistically significant at *p*<0.005 in comparison to group IV (control)

- concentrations of circulating IL-6 levels in healthy subjects were below the detection threshold.

### Cytokine production by blood leukocytes and expression of TLR4 on monocytes- *ex vivo* model

As can be seen from Table 2, blood cells of patients from group I were hyperreactive in production of all three cytokines examined in comparison to the control group (group IV). The levels of Il-1β, IL-6 and TNFα were the highest in group I of NAFLD patients (obese with MS). Moreover, a significant influence of metabolic syndrome (MS) on the level of cytokines was seen when we compared group I (obese with MS) to group III (obese but without MS) or group I to group II (lean without MS). However, a small influence of obesity was also seen, when compared group I with group II (non obese). Metformin significantly reduced the level of cytokines examined (IL-1β, IL-6 and TNFα) as seen in Table 3 in which statistical analysis is presented. It can be seen that metformin decreased cytokine level mainly in group I and II so both MS and obesity seemed to be related to high cytokine level which was sensitive to metformin influence.

**Table 2:** Production of proinflammatory cytokines ex vivo by blood leukocytes of patients with NAFLD, induced by LPS, and comparison to healthy control. The influence of metformin (20 μM lub 100 μM). A comparison of statistical significance between groups.

Met μM		I Group	II Group	III Group	IV Group	Statistical significance
		n = 28	n = 23	n = 22	n = 22	between groups
**Met20 LPS**	IL-1β (pg/ml)	882±72	812±15.6	859±133	435±46	I vs IV *p*<0.007
	IL-6 (pg/ml)	755.6±47.7	618±29.4	69±88	410±23.8	I vs II *p*<0.03;
						I vs IV *p*<0.005
	TNFα (pg/ml)	999±62.5	698±80	763±50.3	441±11.7	I vs II *p*<0.0004
						I vs III *p*<0.003
**Met100 LPS**	IL-1β (pg/ml)	1044±93	991±90.0	910±96.0	595±54	I vs IV *p*<0.0002
						I vs IV *p*<0.0002
	IL-6 (pg/ml)	894.1±38.1	721±17.9	722.6±89.9	549±24.3	I vs III *p*<0.045
						II vs IV *p*<0.04
	TNFα (pg/ml)	1 195±75	791±44.4	886±31	782±77.7	I vs II *p*<0.002
						I vs IV *p*<0.0004
**LPS (C)**	IL-1β (pg/ml)	1 399±85	1270±80.7	992±89	685±27.0	I vs III *p*<0.02
						I vs IV *p*<0.0001
	IL-6 (pg/ml)	1218±127.7	899±73.9	781.3±91.8	646±59.0	I vs III *p*<0.009
						I vs IV *p*<0.0005
	TNFα (pg/ml)	2 336.2 ± 342	2075±164.4	1265 ± 86.7	852.6 ±74.0	I vs III *p*<0.02
						I vs IV *p*<0.0007
						II vs III *p*<0.001
						I vs IV *p*<0.0001
						III vs IV *p*<0.01
**Met 100 (C)**	IL-1β (pg/ml)	<10	<6	<4	<4	
	IL-6 (pg/ml)	<10	<4	<4	<4	
	TNFα (pg/ml)	<40	<40	<30	<10	
**Cells (C)**	IL-1β (pg/ml)					
	IL-6 (pg/ml)	<2	<2	<2	<2	
	TNFα (pg/ml)					

Analysis of variance by Kruskall-Wallis method with Dunn’s test for statistical significance.

**Table 3:** A comparison of statistical significance in the middle of the groups.

Cytokine		I Group	II Group	III Group	IV Group
**IL-1β**	Met20/Met100	0.0001	0.001	n.s	n.s
	Met20/LPS (C)	0.0001	0.0001	n.s	n.s
	Met100/LPS (C)	0.0001	0.0001	n.s	n.s
**IL-6**	Met20/Met100	0.0001	0.02	n.s	0.03
	Met20/LPS (C)	0.0001	0.0001	n.s	n.s
	Met100/LPS (C)	0.0001	0.0001	n.s	n.s
**TNFα**	Met20/Met100	0.016	0.0003	0.0004	n.s
	Met20/LPS (C)	0.0001	0.0001	0.001	n.s
	Met100/LPS (C)	0.0001	0.0001	0.001	n.s

n.s—not significant

Heparinized blood was diluted 1:5 with cell culture medium and incubated for 24 h at 37°C, 5% CO_2_ with LPS (100 ng/ml) and two concentrations of metformin (Met). Next cells were centrifuged (300xg, 5 min) and cytokine level was measured as described in Material and Methods.

When expression of TLR4 on monocytes was examined after *ex vivo* treatment with LPS and/or with metformin, a significantly higher TLR4 expression was seen but only after treatment with LPS alone (Table 4). When considering influence of body weight, obesity- characteristic for the groups I and III- correlated with cytokines' level (group I versus group IV and group III versus group IV) (Table 4). Metformin had a weak activity in decreasing expression of TLR4 in all groups (Table 5).

**Table 4:** Expression of TLR4 (mean MFI) on blood monocytes of patients with NAFLD and control subjects treated ex vivo with LPS (100 ng/ml). The influence of metformin (20 μM or 100 μM). A comparison of statistical significance between groups.

	I Group	II Group	III Group	IV Group	Statistical significance
	n = 28	n = 23	n = 22	n = 22	between groups
					I vs II, III, IV; *p*<0.0001
**Met 20 + LPS**	50.6±9.3	34.0±1.3	30.7±3.6	17.9±3.7	II vs IV; *p*<0.0001
					III vs IV; *p*<0.0001
					I vs II, III, IV; *p*<0.0001
**Met 100 + LPS**	38.4±5.0	22.7±3.5	30.1±7.5	19.3±6.4	III vs IV; *p*<0.0001
					II vs III; *p*<0.03
**LPS (C)**	66.7±3.4	48.9±3.3	51.2±5.8	46.8±5.9	I vs II, III, IV; *p*<0.0001

Analysis of variance by Kruskall-Wallis method with Dunn’s test for statistical significance.

**Table 5:** A comparison of statistical significance in the middle of the groups.

	I Group	II Group	III Group	IV Group
**Met20/Met100**	0.042	0.02	n.s	n.s
**Met20/LPS (C)**	0.01	0.0022	0.001	0.0001
**Met100/LPS (C)**	0.001	0.001	0.001	0.0001

n.s not significant

Expression of TLR4 was measured as mean MFI by flow cytometry on monocytes after incubation with 100 ng/ml of LPS and two doses of metformin.

AKG did not influence cytokines' level in all groups of the examined patients with NAFLD (see Table 6 and Table 7) as well as had no influence on the level of TLR4 expression on blood monocytes (Table 8 and Table 9). AKG but at higher dose only (25mM), decreased IL-1β, IL-6 and TNFα in the control group and did not influence cytokines' production in the NAFLD groups. Moreover, AKG induced low levels of TNFα in blood cell cultures mainly in obese patients with MS. This result needs further conformation and examination.

**Table 6:** Production of proinflammatory cytokines ex vivo by blood leukocytes of patients with NAFLD, induced by LPS, and comparison to healthy control. The influence of alpha ketoglutarate (AKG 25 mM or 10mM). A comparison of statistical significance between groups.

AKG mM		I Group	II Group	III Group	IV Group	Statistical significance
		n = 28	n = 23	n = 22	n = 22	between groups
**AKG 10**						I vs III; *p*<0.0007
	IL-1β (pg/ml)	1 162 ± 282	1 686 ± 769	666±117	653±83	I vs IV; *p*<0.0005
						II vs III; *p*<0.01
						II vs IV; *p* < .009
						I vs II; *p*<0.0005
	IL-6 (pg/ml)	1207±260	696±54	804±109	638 ± 32.7	I vs III; *p*<0.001
						I vs IV; *p*<0.0001
						I vs IV; *p*<0.01
	TNFα (pg/ml)	1686±769	2292±185	1075±560	667±23	II vs III; *p*<0.01
						II vs IV; *p*<0.001
**AKG 25**						I vs IV; *p*<0.0001
	IL-1β (pg/ml)	1044±203	1 162 ± 59	1024±183	439 ± 96	II vs IV; *p*<0.0003
						III vs IV; *p*<0.0002
	IL-6 (pg/ml)	1 260 ± 197	641±90,6	734±63	882 ± 34.2	I vs II, III; *p*<0.0001
						I vs IV; *p*<0.0002
						I vs II; *p*<0.02
						I vs IV; *p*<0.0003
	TNFα (pg/ml)	1 548 ± 504	2326±380	1553±420	475±108	II vs III; *p*<0.04
						II vs IV; *p*<0.0001
						III vs IV; *p*<0.0009
**LPS (C)**						I vs III, IV; *p*<0.0001
	IL-1β (pg/ml)	1 399 ± 85	1270±80.7	992 ± 89	685 ± 27.0	II vs III *p*<0.0004
						II vs IV; *p*<0.0001
						III vs IV; *p*<0.0002
						I vs II; *p*<0.0002
	IL-6 (pg/ml)	1218±127.7	899±73.9	781.3 ± 91.8	646 ± 59.0	I vs III, IV; *p*<0.0001
						II vs IV; *p*<0.0001
						I vs III, IV; *p*<0.0001
	TNFα (pg/ml)	2 336.2 ± 342	2075±164.4	1265 ± 86.7	852.6 ± 74.0	II vs III; *p*<0.0003
						II vs IV; *p*<0.0001
						III vs IV; *p*<0.04
**AKG25 (C)**	IL-1β (pg/ml)	<2	<10	<5	<2	
	IL-6 (pg/ml)	<40	<20	<5	<2	
	TNFα (pg/ml)	156±13.6	137±10.6	29.6±6.5	4.0±1.0	
**Cells (C)**	IL-1β (pg/ml)					
	IL-6 (pg/ml)	<2	<2	<2	<2	
	TNFα (pg/ml)					

Analysis of variance by Kruskall-Wallis method with Dunn’s test for statistical significance.

**Table 7:** A comparison of statistical significance in the middle of groups.

Cytokine		I Group	II Group	III Group	IV Group
**IL-1β**	AKG10/AKG25	n.s	n.s	0.02	0.04
	AKG10/LPS(C)	n.s	n.s	0.02	n.s
	AKG25/LPS(C)	n.s	n.s	n.s	0.04
**IL-6**	AKG10/AKG25	n.s	n.s	0.04	0.04
	AKG10/LPS(C)	n.s	n.s	n.s	n.s
	AKG25/LPS(C)	n.s	n.s	n.s	0.04
**TNFα**	AKG10/AKG25	n.s	n.s	n.s	0.04
	AKG10/LPS(C)	n.s	n.s	n.s	0.04
	AKG25/LPS(C)	n.s	n.s	0.04	0.04

n.s not significant.

Heparinized blood was diluted 1:5 with cell culture medium and incubated for 24 h at 37°C, 5% CO_2_ with LPS (100 ng/ml) and two concentrations of AKG. Next cells were centrifuged (300xg, 5 min) and cytokine level was measured as described in Material and Methods.

**Table 8:** Expression of TLR4 (mean MFI) on blood monocytes of patients with NAFLD and control subjects treated ex vivo with LPS (100 ng/ml). The influence of alpha ketoglutarate (AKG 10 mM or 25 mM). A comparison of statistical significance between groups.

AKG Mm	I Group	II Group	III Group	IV Group	Statistical significance
	n = 28	n = 23	n = 22	n = 22	between groups
					I vs III; *p*<0.03
**AKG 10**	66.2±17.2	46.9±18.8	46.3±2.8	48.5±4.9	I vs III; *p*<0.0004
					I vs IV; *p*<0.0001
**AKG 25**	57.7±23.2	56.2±1.1	41.0±15.6	45.1±1.7	n.s.
**LPS (C)**	66.7±3.4	48.9±3.3	51.2±5.8	46.8±5.9	I versus II, III, IV; *p*<0.0001

Analysis of variance by Kruskall-Wallis method with Dunn’s test for statistical significance.

**Table 9:** A comparison of statistical significance in the middle of the groups.

	I Group	II Group	III Group	IV Group
**AKG10/AKG25**	n.s	n.s	n.s	0.04
**AKG10/LPS(C)**	n.s	n.s	n.s	0.04
**AKG25/LPS(C)**	n.s	n.s	0.04	0.04

n.s not significant

Expression of TLR4 was measured as mean MFI by flow cytometry on monocytes after incubation with 100 ng/ml of LPS and two doses of AKG.

In contrast to that, PC significantly reduced the level of IL-1β, IL-6 and TNFα in blood leukocytes of patients from groups I and III but not from group II. This strongly indicates the role of obesity in high cytokines' response and the role of PC in attenuation of this hyperreactivity (Table 10 and Table 11). The similar influence of obesity was observed when TLR4 expression on blood monocytes was measured by flow cytometry. PC significantly decreased the TLR4 level in group I and III but not in group II thus indicating also the role of obesity in high cytokines' response *ex vivo* to LPS treatment (Table 12 and Table 13).

**Table 10:** Production of proinflammatory cytokines ex vivo by blood leukocytes of patients with NAFLD, induced by LPS, and comparison to healthy control. The influence of PC (PC 20 μg/ml or 100 μg/ml). A comparison of statistical significance between groups.

		I Group	II Group	III Group	IV Group	Statistical significance
		n = 28	n = 23	n = 22	n = 22	between groups
**PC 20**	IL-1β (pg/ml)	598±210	562±183	606±197	659±302	n.s
**LPS**	IL-6 (pg/ml)	807±219	730±87	797±84	946±41	n.s
	TNFα (pg/ml)	770±391	387±259	444±44	954±79	n.s
**PC 100**	IL-1β (pg/ml)	710±210	597±95	434±45	702±220	I vs III; *p*<0.03
**LPS**	IL-6 (pg/ml)	824±188	670±77.3	795±48	934±44	n.s
	TNFα (pg/ml)	831±378	420±379	596±423	966±28	n.s
						I vs III; *p*<0.001
						I vs IV; *p*<0.0001
	IL-1β (pg/ml)	1 399±85	1270±80.7	992±89	685±27.0	II vs III; *p*<0.0004
						II vs IV; *p*<0.0001
						III vs IV; *p*<0.0002
						I vs II; *p*<0.0002
**LPS (C)**	IL-6 (pg/ml)	1218±127.7	899±73.9	781.3±91.8	646±59.0	I vs III, IV; *p*<0.0001
						II versus IV; *p*<0.0001
						I vs III, IV; *p*< 0.0001
	TNFα (pg/ml)	2 336.2±342	2075±164.4	1265±86.7	852.6±74.0	II vs III; *p*<0.0003
						II vs IV; *p*<0.0001
						III vs IV; *p*<0.04
**PC100 (C)**	IL-1β (pg/ml)	<1	<1	<2	<3	
	IL-6 (pg/ml)	<2	<2	<3	<3	
	TNFα (pg/ml)	<20	<10	<20	<4	
**Cells (C)**	IL-1β (pg/ml)	<2	<2	<2	<2	
	IL-6 (pg/ml)					
	TNFα (pg/ml)					

n.s not significant. Analysis of variance by Kruskall-Wallis method with Dunn’s test for statistical significance.

**Table 11:** A comparison of statistical significance in the middle of groups.

Cytokine		I Group	II Group	III Group	IV Group
**IL-1β**	PC20/PC100	n.s	n.s	0.04	n.s
	PC20/LPS(C)	0.001	n.s	0.04	n.s
	PC100/LPS(C)	0.001	n.s	0.04	n.s
**IL-6**	PC20/PC100	n.s	n.s	n.s	n.s
	PC20/LPS(C)	0.004	n.s	n.s	0.04
	PC100/LPS(C)	0.004	n.s	n.s	0.04
**TNFα**	PC20/PC100	n.s	n.s	0.04	n.s
	PC20/LPS(C)	0.004	n.s	0.04	n.s
	PC100/LPS(C)	0.004	n.s	0.04	0.04

n.s not significant

Heparinized blood was diluted 1:5 with cell culture medium and incubated for 24 h at 37°C, 5% CO_2_ with LPS (100 ng/ml) and two concentrations of AKG. Next cells were centrifuged (300xg, 5 min) and cytokine level was measured as described in Material and Methods.

**Table 12:** Expression of TLR4 (mean MFI) on blood monocytes of patients with NAFLD and control subjects treated ex vivo with LPS (100 ng/ml). The influence of PC (20 μg/ml or 100 μg/ml). A comparison of statistical significance between groups.

PC μg/ml	I Group	II Group	III Group	IV Group	Statistical significance
	n = 28	n = 23	n = 22	n = 22	between groups
					I vs II; *p*<0.0001
**PC 20 LPS**	35.5±3.3	40.2±0.5	24.0±2.1	27.9±8.8	I vs III; *p*<0.01
					I vs IV; *p*<0.03
					II vs IV; *p*<0.009
**PC 100 LPS**	30.5±7.5	30.7 ± 9.5	21.6±0.5	24.5±2.1	I vs II, III, IV; *p*<0.0001
					II vs III, IV; *p*<0.0001
**LPS (C)**	66.7±3.4	48.9±3.3	51.2±5.8	46.8±5.9	I vs II, III, IV; *p*<0.0001

Analysis of variance by Kruskall-Wallis method with Dunn’s test for statistical significance.

**Table 13:** A comparison of statistical significance in the middle of the groups.

	I Group	II Group	III Group	IV Group
**PC20/PC100**	n.s	n.s	0.04	n.s
**PC20/LPS (C)**	0.004	n.s	0.04	n.s
**PC100/LPS(C)**	0.004	n.s	0.04	0.04

n.s not significant

Expression of TLR4 was measured as mean MFI by flow cytometry on monocytes after incubation with 100 ng/ml of LPS and two doses of PC.

## Discussion

NAFLD is a multifactorial disease that is related to insulin resistance (IR) and genetic predisposition. In the pathogenesis of NAFLD, a double-hit hypothesis has been proposed, where the first hit includes lipid accumulation in the liver, followed by a second hit in which proinflammatory mediators induce inflammation, hepatocellular injury and fibrosis. But the underlying molecular mechanisms are incompletely understood. Nowadays, a multiple parallel hits hypothesis has been also suggested. It includes genes, lipotoxic agents, endoplasmic reticulum stress, role of oxidative stress, inflammation and adipokines as well as gut-liver axis. Among these factors, interactions between intestinal mucosal barrier and liver, also named as gut-liver axis, have been widely investigated in the pathogenesis of NAFLD. Normally, translocation of gut bacteria or its products to extraintestinal space is effectively prevented by the defense mechanisms including complete intestinal barrier function and cleansing function of the liver. However, disruption of these defense mechanisms can lead to bacterial translocation and to aberrant activation of immune system, which can trigger harmful inflammations in the liver [23].

NAFLD is associated with activation of the innate immune system resulting in chronic sub-clinical inflammation, which particularly affects the adipose tissue and the liver. Recently, bacterial LPS has been implicated as a potent inducer of inflammation. It activates the innate immune pathway via stimulation of Toll-like receptor, especially TLR4, which is the receptor for LPS. Serum LPS levels are increased in patients with hepatic steatosis [24,25]. Circulating LPS levels are elevated also in the majority of animal models of NAFLD induced by diets. Even in mice on standard laboratory chow, continuous subcutaneous infusion of low-dose LPS results in hepatic steatosis, hepatic insulin resistance and hepatic weight gain. These data indicate that the liver is the main target for LPS and TLR4 is the most important receptor playing role in progression of NAFLD. Recently, the main role of liver Kupffer cells (KCs) in LPS-induced imbalance in the oxidative stress and certain pro-inflammatory cytokines and chemokines production was documented [8]. KCs, which could participate in the redistribution of hepatic lymphocyte subsets during NAFLD constitute 20–25% of all non-parenchymal cells in the liver. KCs are the primary source of hepatic pro-inflammatory cytokines, such as TNFα. In addition to their essential role as phagocytes, KCs participate in liver T cell tolerance. Consequently, modified KCs phenotypes could be involved in an altered immune response by disrupting T cell tolerance in the liver [26].

Novel data show that the saturated fatty acid (FAs) palmitic acid (PA) activates the inflammasome and induces sensitization to LPS-induced IL-1β release in hepatocytes. Furthermore, PA triggers the release of danger signals from hepatocytes in a caspase dependent manner. These hepatocyte-derived danger signals, in turn, activate inflammasome, IL-1β, and TNFα release in mononuclear cells of the liver. Saturated FAs represent an endogenous danger in the form of a first hit, up-regulate the inflammasomes, and induce sensitization to a second hit with LPS for IL-β release in hepatocytes. Thus, hepatocytes play a key role in orchestrating tissue responses to danger signals in NAFLD [27]. From our experiments is also seen that not only hepatocytes or KCs but also blood monocytes may be under influence of FAs. Blood monocytes, acting as first hit, become hypersensitive to second hit such as LPS, as the highest levels of proinflammatory cytokines were produced by monocytes of obese patients with high serum level of triglycerides and cholesterol.

The primary role of KCs in NAFLD is considered to be perturbation of the JNK and NF-kB pathways as a result of LPS recognition by TLR4. Moreover, upregulation of CD14 in KCs and hyperreactivity against low-dose LPS were observed in high-fat diet-induced steatosis in mice. Such hyperreactivity was regulated by leptin-mediated signaling indicating the interplay between adipose tissue and liver [3,28]. Other resident liver cells and recruited immune cells also produce many mediators that modulate the status of NAFLD in response to LPS. In our experiments, blood monocytes from patients with NAFLD exhibited significant increase in expression of TLR4 on cells, measured as mean fluorescence intensity (MFI) in flow cytometry. Moreover, LPS *ex vivo* treatment of blood cells additionally increased TLR4 expression. Such increase in TLR4 expression was not connected directly with the obesity or MS, as groups with lean patients and obese without MS also had elevated level of TLR4. Moreover, the monocytes of control group also reacted to LPS with TLR4 enhanced expression.

The majority of chronic liver diseases are accompanied by oxidative stress which induces apoptosis in hepatocytes and liver injury. Recent studies suggest that oxidative stress and insulin resistance (IR) are important in the pathogenesis of NAFLD and in the pathophysiology of diabetic complications. In general, IR is believed to be an important trigger for initiation of NAFLD. However, when comparing overweight to lean NAFLD patients, the latter have minor or no insulin resistance, and appear to have less severe histological disease at presentation. In lean NAFLD patients, peripheral (adipose tissue and skeletal muscles) IR may be more important than hepatic IR. Adipose tissue IR triggers excessive release of fatty acids leading to development of hepatic “lipotoxicity”. The latter, causes that patients with NASH have more severe adipose tissue IR which is independent of body mass. Lean NASH patients may have accelerated lypolysis due to IR, mainly at adipose tissue. Notably, majority of lean NAFLD patients had dyslipidemia, mainly isolated hypertriglyceridemia [29,30].

Metformin has been shown to be hepatoprotective in the insulin-resistant and leptin-deficient ob/ob mouse model of NAFLD. Metformin protects hepatocytes against oxidative stress-induced caspase activation, PARP-cleavage and apoptosis. The anti-apoptotic effect of metformin is in part dependent on HO-1 and bcl-xl induction and inhibition of JNK activation and independent of insulin signaling [31,32].

Metformin is widely used as a major anti-diabetic medicine for the treatment of T2DM. As a drug that primarily targets the liver, metformin suppresses hepatic glucose production and this is the main mechanism by which the drug improves glycemia control in T2DM. Biochemically, metformin suppresses gluconeogenesis and stimulates glycolysis. Metformin also improves insulin resistance and corrects dyslipidemia in patients with T2DM. These beneficial effects of metformin implicate a role for metformin in managing NAFLD. As supported by the results from both human and animal studies, metformin improves hepatic steatosis and suppresses liver inflammation. Mechanistically, the beneficial effects of metformin on hepatic aspects are mediated through both adenosine monophosphate-activated protein kinase (AMPK)-dependent and AMPK-independent pathways [31,33].

Metformin in *ex vivo* study decreased significantly TLR4 expression in all groups of patients and also inhibited production by blood cells of pro-inflammatory cytokines such as IL-1β, IL-6 and TNFα. Our results are in agreement with the results described in other papers [34,35], which detected that metformin suppressed scavengers receptors in mouse macrophages and down-regulated TNFα secretion from mouse and human macrophages.

In contrast to metformin AKG only in control healthy group slightly decreased TLR4 expression and did not influence TLR4 expression in three NAFLD groups. It is known that mitochondria, the main source of oxidative oxygen species (ROS) may trigger steatohepatitis and fibrosis by enhancing the lipid peroxidation and induction of cytokines [36]. Therefore, the effectiveness of several antioxidant agents such as vitamin C, vitamin E, betaine and melatonin, hydroxyl citric acid have been evaluated, as increased levels of antioxidants may serve a protective role against development of NAFLD [36,37]. For example quercetin enhanced level of non-enzymatic and enzymatic antioxidants found in rats with NASH. As AKG, Krebs cycle intermediate possess antioxidative activity, stabilizing redox homeostasis and improves arterial elasticity in aged mice and also modulates the antioxidant levels in rats treated with hepatotoxin [14–16], we decided to examine its influence on TLR4 expression and cytokine production. Unfortunately, AKG only at higher dose used (25mM), decreased IL-1β, IL-6 and TNFα in control group and did not influence cytokine production in the NAFLD groups. Similarly, AKG decreased TLR4 expression on monocytes of control group but not of NAFLD groups.

Phosphatidylcholine (PC; 1, 2-diacyl-sn-glycero-3phosphocholine) is a polyunsaturated fatty acid compound that comprises 45% of plasma and inner mitochondrial membranes, 50% of Golgi membranes and 60% of rough endoplasmic reticulum, nuclear and outer mitochondrial membranes. PC presumably increases the level of serum polyunsaturated fatty acids, particularly linoleic acids in cholesterol esters, and reduces serum TG levels and the serum LDL/HDL ratio. PC decreases TC levels by enhancing cholesterol efflux, down-regulating fatty acid synthesis and increasing cholesterol oxidation to bile salts and significantly decreases TG levels. Furthermore, dietary PC reportedly alleviated orotic acid-induced fatty liver disease through suppression of liver fatty acid synthase activity [38].

In contrast to AKG, in our experiments PC was very effective in attenuation of pro-inflammatory cytokines overproduction in obese patients with NAFLD. A number of recent studies have demonstrated an anti-inflammatory potential of PC in various conditions linked to leukocyte activation including LPS-induced oxidative stress and cytokine production. PC was shown to attenuate serum level of TNFα and IL-6 in mice treated with LPS [12]. Moreover, PC treatment alleviated high fat diet-induced obese status and obesity-related complications such as hyperlipidemic changes that induce NAFLD [11]. Our results strongly suggest that blood leukocytes are also sensitive to PC effect of decreasing the cytokines' level released by these cells in response to LPS. Moreover, this attenuation was seen mainly in leukocytes from obese patients with NAFLD.

## Conclusions

This is the first paper in which NAFLD, obesity and metabolic syndrome separately were considered as factors influencing hyperreactivity of blood leukocytes of patients with NAFLD to *ex vivo* LPS treatment. Additionally, we tried to attenuate that hyperreactivity by using metformin and PC, well known medicaments in liver diseases, obesity and accompanied metabolic syndrome. We confirmed the results of other authors who detected that animals with experimental NAFLD are hypersensitive to LPS treatment and produce increased levels of pro-inflammatory cytokines. In our experiments blood cells from 3 groups of patients with NAFLD were treated ex vivo with LPS and compared to healthy control subjects. Hyperreactivity was characteristic mainly to obese patients with NAFLD together with MS and decreased with the severity of disease. Metformin was the most effective in attenuation of such hyperreactivity in all 3 groups of patients with NAFLD, but in obese patients the effectiveness of metformin was the weakest. The reduction of cytokines' level was accompanied by the decrease in TLR4 expression. PC also attenuated hyperreactivity to LPS but mainly in obese patients. These results indicate that obesity is the state which complicates the effectiveness of metformin. In contrast to metformin PC is most effective in obese patients.

## Abbreviations

NAFLD: nonalcoholic fatty liver disease
MS: metabolic syndrome
TLR4: Toll-like receptor 4
hs-CRP: high-sensitivity C-reactive protein
NASH: nonalcoholic steatohepatitis
KCs: Kupffer cells
FFAs: free fatty acids
T2DM: type 2 diabetes mellitus
AU: abdominal ultrasonography
TG: triglyceride
TC: total cholesterol
ASPAT: aspartate aminotransferase
ALAT: alanine aminotransferase
IR: insulin resistance
HOMA: homeostatic model assessment
FLI: fatty liver index
HSI: hepatic steatosis index
BMI: body mass index
HDL: high-density lipoprotein
LDL: low-density lipoprotein
MFI: mean fluorescence intensity
MHO: metabolically healthy obesity
LPS: lipopolysaccharide
AKG: alpha ketoglutarate
PC: phosphatidylcholine
TNF: tumor necrosis factor-α
FAs: saturated fatty acid
PA: palmitic acid
